# Supplementary material for: FastqPuri: high-performance preprocessing of RNA-seq data
Source: BMC Bioinformatics. 2019 May 3;20:226. doi: 10.1186/s12859-019-2799-0 (PMC6500068; doi:10.1186/s12859-019-2799-0)
Supplement: Supplementary file 2 — Archive of FastqPuri. Archive containing all files needed to install and run FastqPuri v1.0.6. Date stamp March 22, 2019. (GZ 47,819 kb) [file 12859_2019_2799_MOESM2_ESM.gz › FastqPuri-1.0.6/html/init__makeBloom_8h.html]

FastqPuri: include/init\_makeBloom.h File Reference


FastqPuri


- include

Classes |
Typedefs |
Functions

init\_makeBloom.h File Reference

Help dialog for makeBloom and initialization of the command line arguments.
More...

`#include <stdint.h>`  
`#include "defines.h"`

Include dependency graph for init\_makeBloom.h:

This graph shows which files directly or indirectly include this file:

Go to the source code of this file.

|  |  |
| --- | --- |
| Classes | |
| struct | \_iparam\_makeBloom |
|  | contains makeBloom input parameters More... |
|  | |

|  |  |
| --- | --- |
| Typedefs | |
| typedef struct \_iparam\_makeBloom | Iparam\_makeBloom |
|  | contains makeBloom input parameters More... |
|  | |

|  |  |
| --- | --- |
| Functions | |
| void | printHelpDialog\_makeBloom () |
|  | Function that prints makeBloom help dialog when called. |
|  | |
| void | getarg\_makeBloom (int argc, char \*\*argv) |
|  | Reads in the arguments passed through the command line to makeBloom. and stores them in the global variable par\_MB. |
|  | |

## Detailed Description

Help dialog for makeBloom and initialization of the command line arguments.

Author
:   Paula Perez paula.nosp@m.pere.nosp@m.zrubi.nosp@m.o@gm.nosp@m.ail.c.nosp@m.om

Date
:   05.09.2017

## Typedef Documentation

## ◆ Iparam\_makeBloom

typedef struct \_iparam\_makeBloom Iparam\_makeBloom

contains makeBloom input parameters

Note
:   nelemen will be computed once the fasta file is read and loaded.


---

Generated on Mon Mar 19 2018 23:42:01 for FastqPuri by  

 1.8.14
